# Detection of Portal Venous Gas by Point-of-Care Ultrasound in Severe Abdominal Disease: A Case Series

**DOI:** 10.7759/cureus.100153

**Published:** 2025-12-26

**Authors:** Matthew Kongkatong, Gitansh Bhargava, Jakob Ottenhoff, James Moak, Christopher Thom

**Affiliations:** 1 Emergency Medicine, University of Virginia School of Medicine, Charlottesville, USA; 2 Emergency Medicine, University of Arkansas for Medical Sciences, Little Rock, USA

**Keywords:** abdominal emergency, intestinal pneumatosis, pocus, point-of-care ultrasound, portal venous gas, pvg

## Abstract

Portal venous gas (PVG) is an uncommon but critical finding for emergency physicians (EPs) to recognize on point-of-care ultrasound (POCUS). Gas bubbles appear as hyperechoic foci flowing in the proximal vasculature and may appear as linear structures in the distal veins. This series describes three patients presenting with complaints of abdominal pain in whom POCUS examination identified PVG. In all three cases, serious abdominal pathology (one case of acute mesenteric ischemia and two cases of necrotizing intestinal infection) was confirmed with additional workup. PVG is associated with serious abdominal pathology, and although it is not pathognomonic for mesenteric ischemia, this condition remains the most frequent underlying cause of PVG. Mortality among patients with PVG varies but can be very high. EPs should familiarize themselves with the appearance of PVG on POCUS and remain vigilant for this finding while evaluating their patients.

## Introduction

Point-of-care ultrasound (POCUS) is a powerful bedside tool to expedite the evaluation of abdominal complaints in the emergency department (ED) [1]. Though rare, portal venous gas (PVG) may be found while scanning patients with abdominal complaints. Gas has low acoustic impedance compared to tissues and produces highly reflective interfaces. These interfaces are hyperechoic and produce reverberation artifacts in the case of a large gas bubble, whereas tetrahedrons of small gas bubbles produce a ring-down artifact. A ring-down artifact will appear as a continuous echogenic band of reverberations deep to a hyperechoic focus of air. This is in contrast to a comet-tail artifact, where the reverberations decrease in amplitude shortly after their origin. Comet-tail artifacts originate from closely spaced reflective interfaces such as calcium or cholesterol crystals, whereas ring-down artifacts are specific for air [2]. 

Two mechanisms have been proposed to explain the presence of gas in the portal venous system. One explanation is that increased bowel intraluminal pressure, combined with loss of mucosal integrity, can lead to absorption of gas into the mesenteric vasculature. The other is that PVG can originate from a gas-producing microbial infection that causes mucosal damage to the gastrointestinal system. Thus, previous literature has associated PVG with potentially life-threatening conditions such as gastrointestinal ischemia, obstruction, and perforation [3,4]. The use of POCUS in the evaluation of ill-appearing patients with abdominal pain can allow emergency physicians (EPs) to identify high-risk patients when PVG is found. Efforts should then turn to determining the underlying cause with computed tomography (CT) or CT angiogram (CTA). Management of the underlying conditions that commonly cause PVG may include surgical intervention, so early surgical consultation is warranted. Herein, we describe three patient presentations in whom POCUS examination rapidly identified PVG.

## Case presentation

This case series was exempt from institutional review board (IRB) review as it did not meet the definition of human subjects research as stated in 45 Code of Federal Regulations (CFR) 46.202. All three cases presented to an academic ED in a suburban/rural setting between 2022 and 2023 (Table 1). 

**Table 1:** Summary of the three cases POCUS: point-of-care ultrasound; PVG: portal venous gas

Variable	Case 1	Case 2	Case 3
Sex	Female	Male	Male
Age	57 years	71 years	67 years
Race	Caucasian	Caucasian	African American
Comorbidities	Gastric carcinoma	Type 2 diabetes, chronic kidney disease stage 3, coronary artery disease	Chronic kidney disease stage 5, atherosclerosis, ischemic cardiomyopathy, hypertension
POCUS findings	Focal hyperechoic signals in the peripheral liver parenchyma, hyperechoic foci within the portal vein, colonic pneumatosis	Focal hyperechoic signals in the peripheral liver parenchyma, hyperechoic foci within the portal vein, large volume ascites with focal loculation	Focal hyperechoic signals in the peripheral liver parenchyma, hyperechoic foci within the portal vein
Etiology of PVG	Necrotizing intestinal infection	Necrotizing intestinal infection	Acute on chronic mesenteric ischemia
Surgical intervention	Partial colectomy	Not offered	Exploratory laparoscopy
Outcome	Survived to hospital discharge	Death due to septic shock	Survived to hospital discharge

Case 1

**Figure 1:**
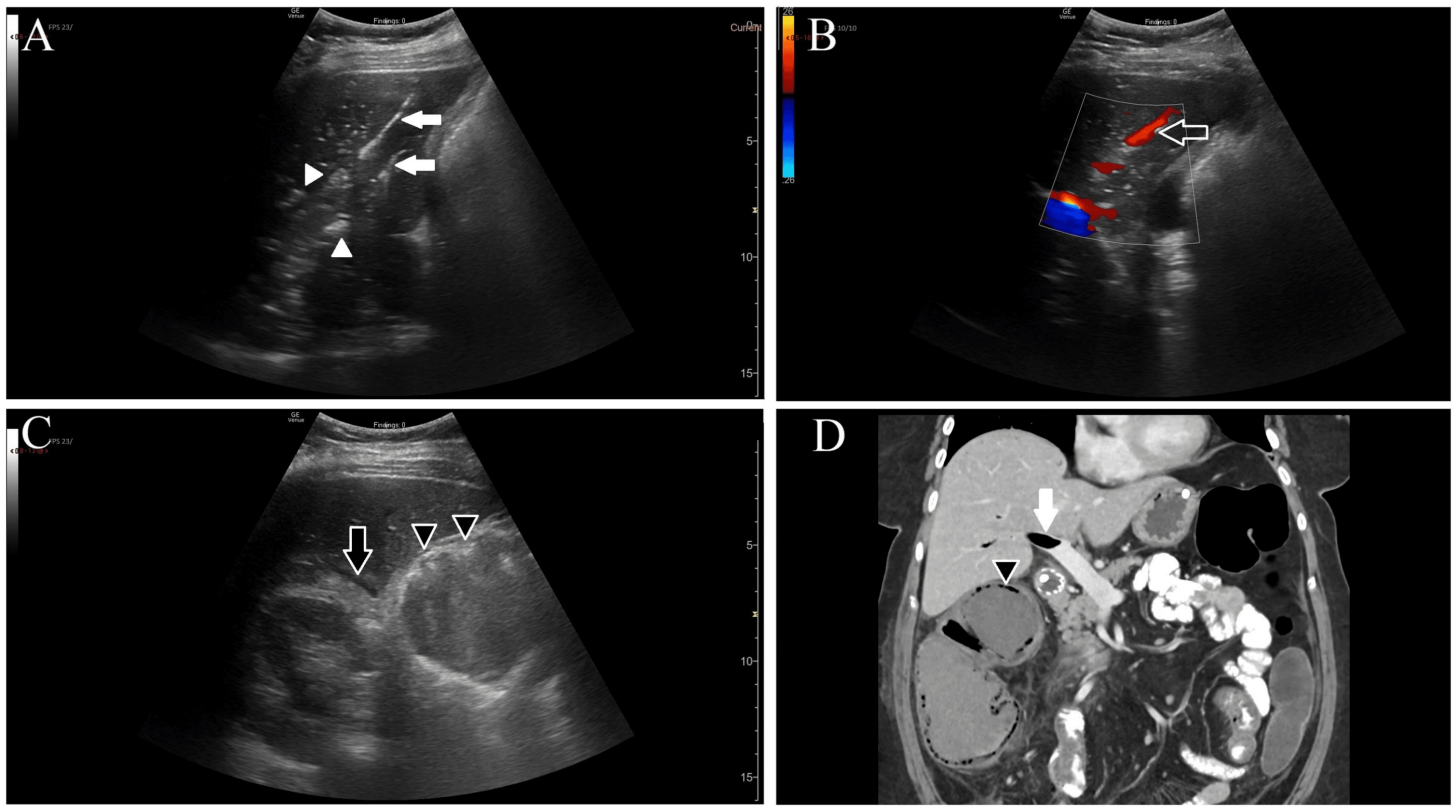
Case 1 - POCUS images and CT A: Hyperechoic collections of PVG (white arrowheads) are visualized in the liver parenchyma. Linear collections of gas (white arrows) are seen within portal veins coursing in the long-axis plane. B: Color Doppler imaging of the linear collection (black arrow) demonstrates flow within the structure, identifying it as a portal vein containing microbubbles. C: B-mode image demonstrates perihepatic free fluid (black arrow) and colonic pneumatosis (black arrowheads). D: CT image demonstrates gas (white arrow) within the portal vein and colonic pneumatosis (black arrowhead). B-mode: brightness mode; PVG: portal venous gas; POCUS: point-of-care ultrasound; CT: computed tomography

A 57-year-old female patient with a history of gastric cancer presented to the ED for three days of fatigue, abdominal pain, and vomiting. Her vital signs were: temperature 36.7°C (98.1°F), heart rate 141 beats per minute, blood pressure 124/75 mmHg, respiratory rate 18 breaths per minute, and SpO_2_ 95%. She was ill-appearing and had severe right upper quadrant (RUQ) abdominal tenderness with guarding. She was treated with 2 g of cefepime per the institution's sepsis protocol for cancer patients. After initial assessment by the primary ED team, a POCUS examination of the RUQ of the abdomen was performed using a low-frequency (1.4-5.7 MHz) curvilinear probe (GE, Chicago, IL, USA) by a separate attending EP with a focused practice designation in advanced emergency ultrasonography while leading a teaching session for medical students. Patchy, hyperechoic foci were visualized within the liver parenchyma along with linearly organized hyperechoic structures with ring-down artifact (Figures 1A-1B). Color Doppler showed flow corresponding to the linear structures. Trace perihepatic fluid and a round, fluid-filled structure, 6 cm in diameter with a hyperechoic rim, were also visualized in the RUQ, representing colonic pneumatosis intestinalis (Figure 1C). These findings were interpreted as representing PVG along with bowel dilatation and were verbally conveyed to the treating attending EP. They ordered an abdominopelvic CT, which was performed four hours later, showing PVG and colitis with mucosal wall pneumatosis (Figure 1D). General surgery consultation recommended treating the patient with 4.5 g of intravenous piperacillin/tazobactam and operative management of bowel ischemia. They performed an emergency laparotomy and identified right colon necrosis necessitating a hemicolectomy with end ileostomy and mucus fistula formation. Postoperatively, she was admitted to the surgical ICU for septic shock and acute blood loss but recuperated and was discharged 11 days later.

Case 2

**Figure 2:**
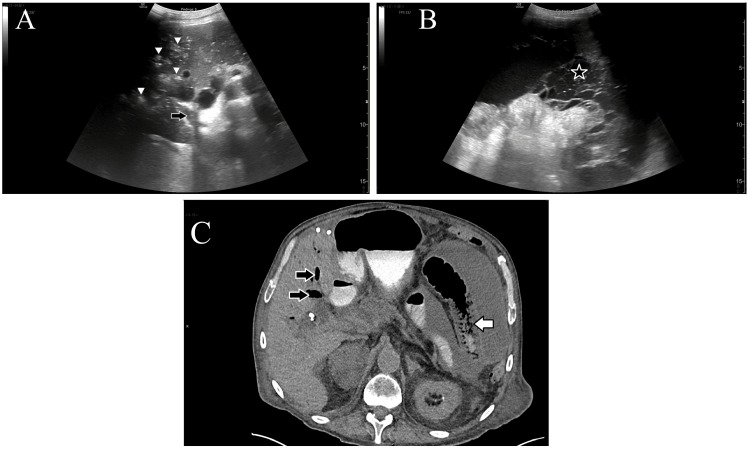
Case 2 - POCUS images and CT A: B-mode image of the liver shows collections of hyperechoic gas (white arrowheads) within the parenchyma, as well as gas (black arrow) flowing within the portal vein. B: A loculated fluid collection (star) is visible in the upper abdomen. C: CT image demonstrates dilated small bowel (white arrow) with mucosal sloughing and gas (black arrows) within the portal veins. B-mode: brightness mode; POCUS: point-of-care ultrasound; CT: computed tomography

A 71-year-old male patient with a history of pancreatitis and chronic cholecystitis complicated by previous perforation presented to the ED for one week of worsening fatigue, vomiting, and right-sided abdominal pain. His vital signs were: temperature 36.4°C (97.6°F), heart rate 70 beats per minute, blood pressure 116/78 mmHg, respiratory rate 30 breaths per minute, and SpO_2_ 98%. Abdominal examination was notable for distention with a fluid wave but minimal tenderness. A POCUS examination of the RUQ of the abdomen was performed using a low-frequency (1.4-5.7 MHz) curvilinear probe (GE, Chicago, IL, USA) by a separate attending EP with a focused practice designation in advanced emergency ultrasonography while leading a teaching session for medical students. It showed patches of hyperechoic foci within the liver parenchyma with ring-down artifact, as well as flowing echogenic foci within the portal vein (Figure 2A). Loculated free fluid was visualized in the mid-abdomen (Figure 2B). These findings were verbally reported to the treating attending EP. A CT of the abdomen and pelvis showed jejunal edema, mucosal sloughing with associated mesenteric, and PVG (Figure 2C). It also demonstrated gangrenous cholecystitis and necrotizing pancreatitis. He was treated with 4.5 g of intravenous piperacillin/tazobactam and admitted to the ICU for septic shock. He was evaluated by general surgery and felt to be too high-risk for surgical treatment of necrotizing enteritis. A follow-up abdominopelvic CTA after ICU admission demonstrated diminutive distal mesenteric arteries with jejunal ischemia in addition to the initial findings. His gangrenous cholecystitis was managed with a percutaneous cholecystostomy drainage tube. He did not respond to aggressive treatment, developing renal failure, polymicrobial bacteremia, and encephalopathy. He was transferred to the inpatient hospice, where he later died of septic shock.

Case 3

**Figure 3:**
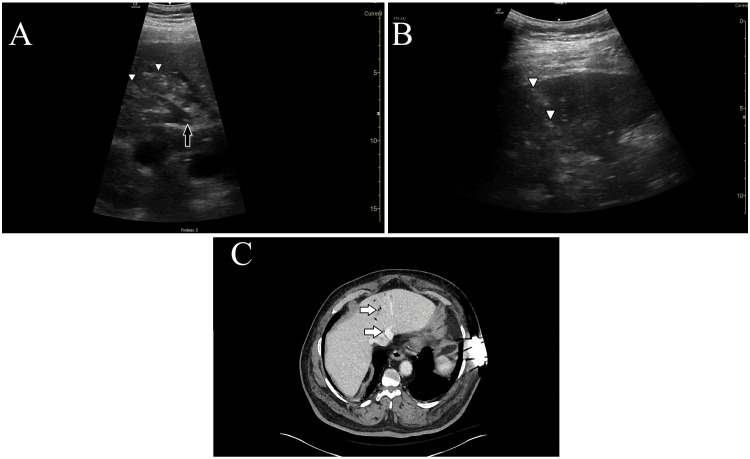
Case 3 - POCUS images and CT A: Narrow-sector B-mode image shows hyperechoic foci of gas (white arrowheads) within the liver, with flowing bubbles (black arrow) visible in the portal vein. B: Full-sector B-mode image shows hyperechoic foci of gas (white arrowheads) within the liver parenchyma. C: CT image demonstrates gas (white arrows) within the portal veins. B-mode: brightness mode; POCUS: point-of-care ultrasound; CT: computed tomography

A 67-year-old male patient with a history of diabetes, hypertension, peripheral arterial disease, and renal failure presented to the ED with a complaint of sudden-onset abdominal pain. His vital signs were: temperature 36.3°C (97.4°F), heart rate 81 beats per minute, blood pressure 190/77 mmHg, respiratory rate 20 breaths per minute, and SpO_2_ 97%. He appeared to be in severe pain with diffuse abdominal distention and epigastric tenderness to palpation without rigidity. A POCUS examination of the RUQ of the abdomen using a low-frequency (1.4-5.7 MHz) curvilinear probe (GE, Chicago, IL, USA) was performed by the treating attending EP with a focused practice designation in advanced emergency ultrasonography. The exam identified hyperechoic foci within the liver parenchyma and similar foci in motion within the portal vein (Figures 3A-3B). These findings were suspicious for PVG. An abdominopelvic CTA showed findings of colonic pneumatosis and PVG (Figure 3C) in addition to severe, multifocal mesenteric arterial stenoses. Given the abruptness of symptom onset, the patient was suspected of having acute mesenteric ischemia. The patient was treated with 2.25 g of intravenous piperacillin/tazobactam and evaluated by general surgery. Emergent diagnostic laparoscopy was performed, which did not show necrotic bowel, and he was admitted to the surgical intermediate care unit. The patient was discharged 10 days later after tolerating the advancement of diet and experiencing improvement in pain. On a subsequent ED presentation, 12 days later, a CT of the abdomen showed resolution of the colonic pneumatosis and PVG. 

## Discussion

These case presentations contribute to the emerging body of literature highlighting the importance of recognizing PVG on POCUS examinations of ED patients. In all three cases, the POCUS examination rapidly discovered PVG secondary to serious abdominal pathology before CT. In cases 1 and 2, there was a several-hour delay between the ordering and completion of the CT. In case 3, the information gained from the POCUS examination led the treating team to expedite the CT study, which minimized time to surgical consultation (Table 2).

**Table 2:** Summary of imaging test completion times and management (24-hour format) ^+^A priority 2 CT was initially ordered at 18:55 but was changed to a priority 1 examination at 19:25. *Operative case start was the following day. POCUS: point-of-care ultrasound; CT: computed tomography; OR: operating room; N/A: not applicable

Time	Case 1	Case 2	Case 3
Roomed	13:00	12:41	17:20
POCUS exam start	14:15	13:41	19:15
POCUS exam end	14:33	13:53	19:20
CT ordered	14:56	14:22	19:25^+^
CT images available	19:23	16:57	20:05
CT read by radiology	19:42	18:02	20:54
Surgery consultation	19:50	18:43	20:21
OR case start	23:05	N/A	07:45*

POCUS has been shown to decrease time to diagnosis and length of stay for other abdominal conditions. A 2024 retrospective study of the impact of POCUS on the diagnosis of small bowel obstruction conducted by Di Gioia et al. showed a median time to diagnosis of 96 minutes faster in the POCUS group versus the CT-only group, as well as a shorter ED length of stay [5]. Huang et al. showed a shorter ED length of stay and decreased time to surgical consultation in patients diagnosed with acute cholecystitis on POCUS performed within 60 minutes of ED presentation [6]. POCUS has been shown to decrease the time to diagnosis of time-sensitive conditions like ascending aortic dissection [7]. In a similar way, the identification of PVG on POCUS could decrease the time to diagnosis of the pathological conditions that cause it. 

Numerous etiologies of PVG have been reported in the literature, including intestinal ischemia, bowel inflammation/infection, mechanical obstruction, post-procedural, and rarer causes such as decompression illness and hydrogen peroxide ingestion [3]. In a retrospective review of published literature, Hussain et al. found that intestinal ischemia, either from acute occlusion or bowel hypoperfusion, is the most frequent etiology of PVG in adults [4]. A single-center 10-year retrospective review of 164 patients performed by Daneshmand et al. in 2020 reported intestinal ischemia as the cause of PVG in nearly 43% of cases. Inflammatory conditions (22%) and mechanical obstruction (17%) represented the second and third most common causes [8]. A larger five-year retrospective review of the National Japanese Inpatient Database (N=1590) performed by Koizumi et al. in 2018 found a similar percentage of bowel ischemia (53%) as the cause of PVG [9]. Mortality rates in patients with PVG around the time of initial description were reported to be around 75%. Present data suggest variable mortality dependent on the underlying etiology of the PVG rather than the PVG finding itself; these are summarized in Table 3 [8,9]. Thus, although not a harbinger of death, the presence of PVG should prompt the EP to be highly concerned about a serious abdominal condition.

**Table 3:** Incidence and mortality rates of different etiologies of PVG *Mortality rates were combined in the original study. PVG: portal venous gas

Data source	Etiology	Patients	Mortality
Daneshmand et al. 2020, single-center retrospective chart review [8]	Ischemia	70/164 (42.68%)	51/70 (72.8%)
	Obstruction	29/164 (17.68%)	15/29 (51.7%)
	Inflammatory/infectious conditions	37/164 (22.56%)	8/37 (21.6%)
	Post-procedural	7/164 (4.27%)	7/28 (14.3%)*
	Other	12/164 (7.32%)	
	Undetermined	9/164 (5.49%)	
Koizumi et al. 2018, national inpatient database retrospective chart review [9]	Ischemia	842/1590 (53.0%)	226/842 (26.8%)
	Obstruction	164/1590 (10.3%)	51/164 (31.1%)
	Inflammatory/infectious conditions	155/1590 (9.7%)	19/155 (12.3%)
	Gastrointestinal ulceration/perforation	87/1590 (5.4%)	15/87 (17.2%)
	Malignancy	56/1590 (3.5%)	19/56 (33.9%)
	Unspecified	286/1590 (18.0%)	104/286 (36.4%)

Depending on the amount of gas present, PVG can appear dramatically on POCUS examination (Table 4). In the above cases, the PVG appeared as hyperechoic areas within the normally hypoechoic liver parenchyma. On occasions, the gas took on a linear configuration in windows where the portal vein was viewed in its long axis. In all cases, gas bubbles were also visible flowing within the portal vasculature near the liver hilum (Video 1). This appearance is characteristic of PVG on brightness mode (B-mode) ultrasound, as described in the literature [10]. Other findings, such as the "meteor shower" sign on motion mode (M-mode) and the "flaming portal vein" sign on Color Doppler mode, have also been described [10,11]. PVG may also feature characteristic "dirty" shadowing typical of air. This finding is distinct from the anechoic and well-demarcated shadowing from calcification [12]. Pneumobilia can have a similar appearance to PVG and may be seen in pathologic states such as cholangitis and fistula formation, as well as non-pathologic states such as after biliary procedures and sphincter of Oddi incompetence. The presence of pneumobilia can persist for years after sphincterotomy [13]. Pneumobilia tends to remain in the proximal biliary tract in contrast with PVG, and a patient's history of biliary instrumentation can aid in differentiation. Consolidated lung with dynamic air-bronchograms can also look similar to PVG, though it should be easily distinguished by its cephalad location relative to the diaphragm. Patients who have recently received a contrast-enhanced ultrasound examination may have delayed clearance of the contrast material, which can appear as hyperechoic foci within the portal vein [14]. 

**Table 4:** Checklist for detecting PVG B-mode: brightness mode; M-mode: motion mode; PVG: portal venous gas Table created by the authors based on information from [10,11].

Probe position	Findings to observe	Comments
Obtain a view of the liver in the midaxillary line	Evaluate liver parenchyma for peripherally-located, non-shadowing hyperechoic foci on B-mode	Calcifications will have anechoic shadowing
		Pneumobilia tends to reside centrally
Obtain a view of the portal vein	Observe for hyperechoic foci within the portal vein flowing in a hepatopetal direction on B-mode	Pneumobilia tends to be static
		Decreasing sector width can increase framerate, making bubble movement easier to appreciate
	May apply Color Doppler imaging to observe for exaggerated Doppler shift artifact caused by PVG (flaming portal vein sign)	-
	May apply M-mode imaging through the portal vein, looking for hyperechoic linear artifacts from PVG (meteor shower sign)	-

**Video 1:** B-mode cine clips, without Color Doppler, show hepatopetal flow of echogenic gas bubbles within the portal vein (arrows) in each case B-mode: brightness mode

## Conclusions

Although a rare entity, PVG is readily visualized by ultrasonography of the liver, including POCUS. In ill-appearing patients presenting with undifferentiated abdominal pain, EPs should consider performing a POCUS examination of the liver to look for PVG. The identification of PVG should both raise suspicion for highly morbid and life-threatening conditions and prompt further workup.
